# Case Report: Chronic pancreatitis in children as the cumulative effect of bilio-pancreatic abnormalities and genetic mutations

**DOI:** 10.3389/fped.2024.1393891

**Published:** 2024-06-21

**Authors:** Francesca Destro, Eleonora Durante, Raffaele Salerno, Alessandro Campari, Milena Meroni, Veronica Diotto, Marco Brunero, Gloria Pelizzo

**Affiliations:** ^1^ Department of Pediatric Surgery, Buzzi Children's Hospital, Milan, Italy; ^2^ Gastroenterology and Digestive Endoscopy Unit, ASST Fatebenefratelli Sacco, Milan, Italy; ^3^ Department of Pediatric Radiology, Buzzi Children's Hospital, Milan, Italy; ^4^ Pediatric Anesthesia and Intensive Care Unit, Buzzi Children's Hospital, Milan, Italy; ^5^ Department of Biomedical and Clinical Sciences, University of Milan, Milan, Italy

**Keywords:** children, chronic pancreatitis, bilio-pancreatic malformation, PRSS1, endoscopy, genetic malformations, anatomic variants, pancreatic duct duplication

## Abstract

Pancreatitis, in general, is a high-morbidity condition. Genetic conditions and anatomic variants are sometimes seen, especially in children, where biliary etiologies and alcohol are less common than in adults. The decision to intervene, the combined operative-endoscopic strategy, and the timing pose unique challenges. We report the case of a 10-year-old boy with PRSS1 mutation and pancreatic duct duplication, discussing the management and reviewing the recent reports in the Literature.

## 1 Introduction

Recurrent (RP) and chronic pancreatitis (CP) are relatively uncommon in pediatric patients. The incidence of pediatric CP is 2 cases out of 100,000 children per year (1). RP and CP are considered a disease continuum together with acute pancreatitis (AP). They are usually associated with genetic mutations, congenital pancreatic abnormalities, and other conditions, such as autoimmune pancreatitis and metabolic disease. Sometimes, the cause of pancreatitis remains unknown (idiopathic forms).

Recent data have demonstrated a high prevalence of genetic mutations among pediatric patients with RP and CP. In particular, mutations that most commonly play a crucial role in the etiology of CP are associated with the PRSS1 (gene-encoding cationic trypsinogen), SPINK1 (Serine Protease Inhibitor Kazal type 1), CFTR (cystic fibrosis), CTRC, and CPA1 genes (2). All these mutations impact pancreatic physiology in different ways. Some authors hypothesized that they may also increase the frequency of some associated anatomical alterations (3). For instance, Bertin et al. found that the frequency of pancreas divisum was higher in patients with genetic pancreatitis, especially in the CFTR group, compared to controls. Similarly, Choudary et al. had a 22% rate of CFTR mutations in patients with asymptomatic pancreas divisum (4).

Anatomical variants may act as causes of pancreatitis, determining an alteration of the pancreatic drainage (e.g., for the presence of narrowing and stones). The accumulation of the exocrine secretion, together with intrahepatic enzyme activation, sustains acute and chronic inflammation and irreversible parenchymal changes (5, 6). This irreversible process destroys the gland, determining fibrosis, acinar and islet cell loss, and progressive loss of exocrine and endocrine functions (6, 7). Pancreas divisum (PD) is the most frequent pancreatic anatomical variation reported in patients with pancreatitis. Other structural abnormalities include extra-pancreatic malformations, such as choledochal cysts, duodenal duplication, and duodenal stenosis (8). Pancreatic duct duplications can cause pancreatitis by the exact mechanism described above.

We present a case report of a 10-year-old boy with CP caused by two contributing factors: a genetic PRSS1 mutation and a duplication of the pancreatic duct. Despite having a family history of PRSS1 mutation (an uncle had total pancreatectomy as an adult, and a brother had pancreatic malformations and biliary tree stones), the boy's several episodes of acute pancreatitis were underestimated. He came to our attention only at the age of 10 years with signs of chronic disease. Further investigations permitted the diagnosis of the PRSS1 mutation and the anatomical variation of the pancreatic duct.

The paper aims to remember the possible association of genetic anomalies with bilio-pancreatic malformations as a possible cause of PD. Attention should be paid to the cumulative effect of these two alterations in the natural history of pancreatitis. The challenges of combined endoscopic and surgical management in the pediatric age are also enlightened.

## 2 Case report

**Figure 1:**
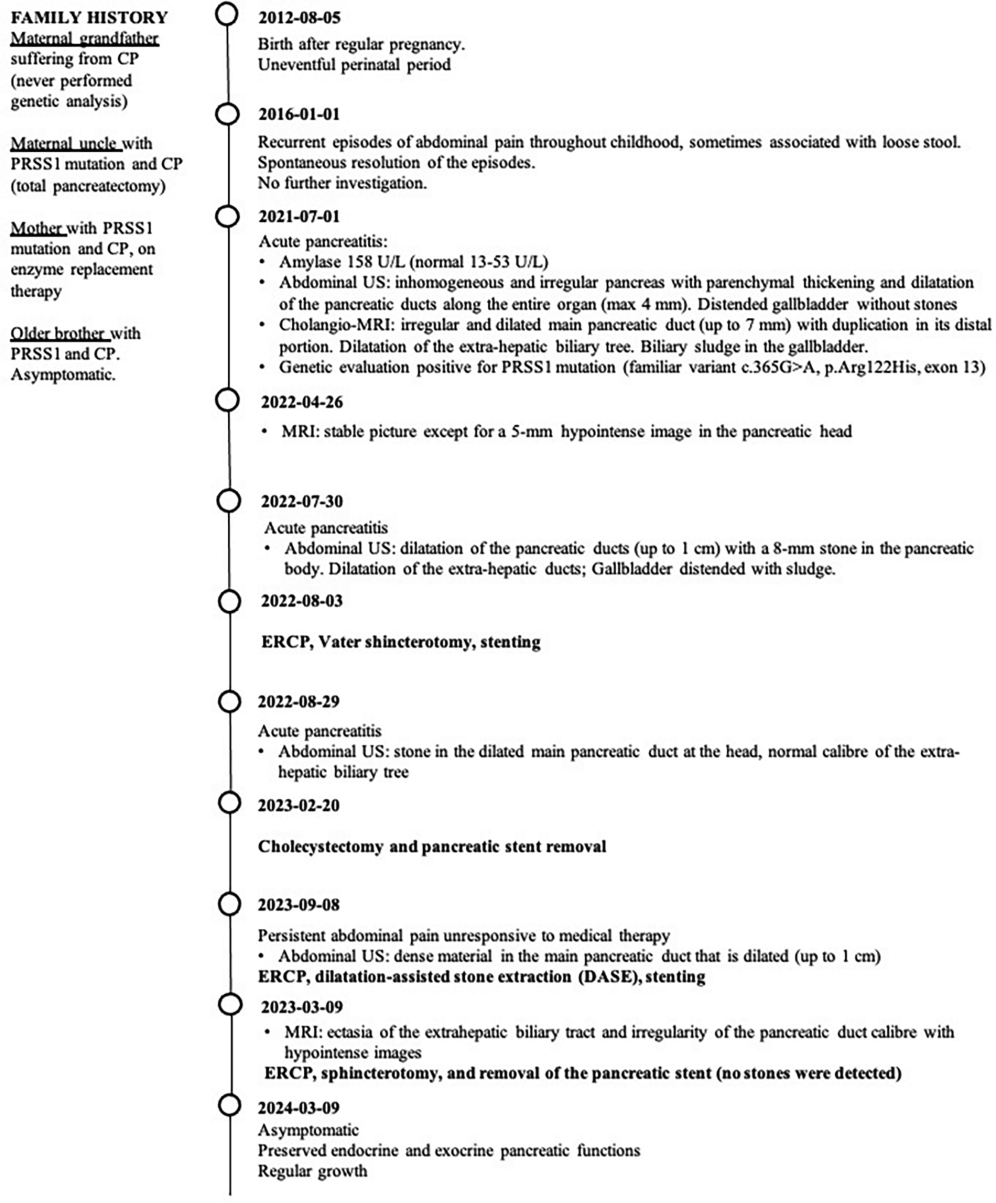
Timeline.

Figure 1 showcases a timeline with relevant data from the episode of care.

A 10-year-old boy came to our attention for recurrent episodes of abdominal pain associated with vomiting and loosestool. He had been complaining of recurrent abdominal pain since early childhood, but he never performed further investigations.

### 2.1 Past medical and family history

He was born on term after a regular pregnancy, and his past medical history was uneventful.

From the maternal side, his grandfather and his two aunts had chronic pancreatitis, but they refused to perform genetic investigations. His uncle was diagnosed with chronic pancreatitis and PRSS1 gene mutation. He required pancreatectomy with Wirsung-jejunal anastomosis in his thirties.

His mother has chronic pancreatitis and PRSS1 mutation diagnosed in adulthood. She is currently taking an enzyme replacement therapy and is followed up conservatively.

His older brother had similar episodes of recurrent abdominal pain at the age of 12 years. Due to the known family history, he underwent some investigations. Abdominal US and MRI showed signs of chronic pancreatitis (a severe adipose involution of the pancreas and an irregular pancreatic duct dilatation) with no evidence of anatomic malformations. Symptomatic drugs were enough to make the clinical picture regress. After 12 months of conservative follow-up, he did not experience any relapse.

**Figure 2:**
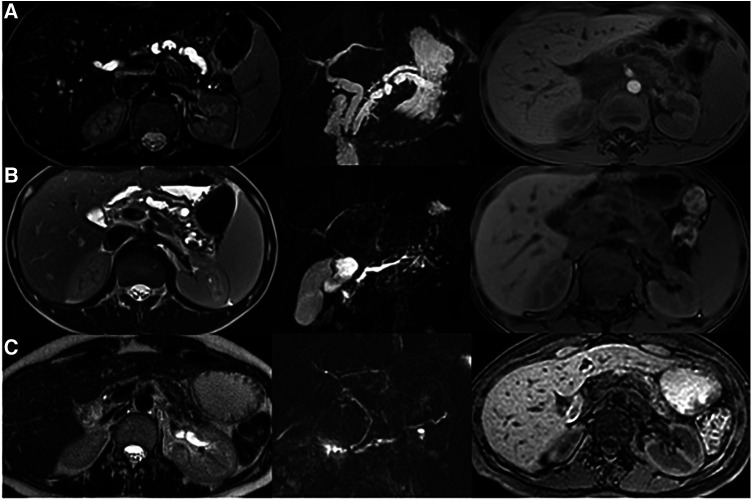
MRI evaluation in family members with PRSS1 mutation. (**A**) Reported case: Ectasia of the main biliary branches (8 mm). Duplication of the main pancreatic duct that appears dilated and irregular. A stenosis (from 9 to 6 mm) is shown in the first portion of the duct. The pancreatic parenchyma has reduced thickness and altered signal. (**B**) Older brother: The main pancreatic duct is serpiginous and dilated up to a maximum caliber of 5 mm. The pancreatic parenchyma has reduced thickness and altered signal. (**C**) Mother: The pancreatic duct is single and of normal caliber. The pancreatic parenchyma is significantly reduced.

Figure 2 compares the imaging of the patient, his mother, and his older brother.

### 2.2 Clinical findings and management

The first admission of the boy was required for acute pancreatitis at the age of 10 years.

The laboratory analysis revealed high amylase levels (158 U/L with a standard range of 13–53 U/L).

The abdominal US showed an inhomogeneous and irregular pancreas with mild parenchymal thickening (approximately 2 cm in the head and 12 mm in the body) and dilation of the pancreatic ducts along the entire organ (4 mm of maximum caliber). The bile ducts appeared dilated (the caliber of the main duct and main intrahepatic branches were 3–4 mm and 2 mm, respectively). The gallbladder appeared to be hugely distended, but there were no indications of either wall thickening or stones.

**Figure 3:**
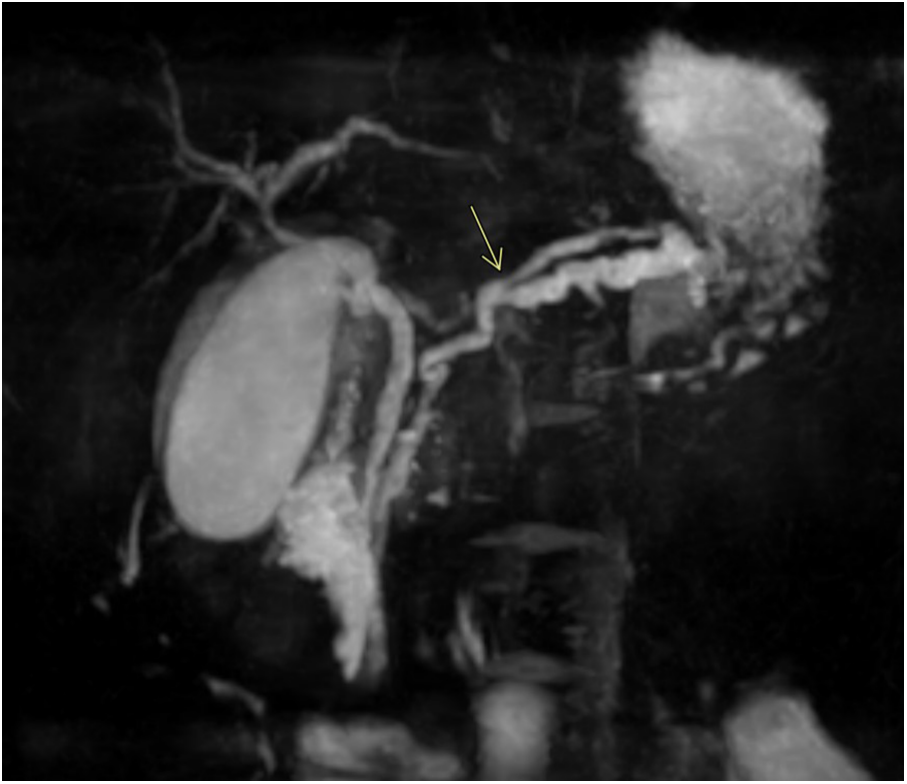
Coronal MIP reformation from the magnetic resonance cholangiopancreatography (MRCP) sequence shows duplication of the main pancreatic duct (arrow). The gallbladder is distended by biliary sludge.

The cholangio-MRI confirmed the presence of an irregular and dilatated main pancreatic duct (Figure 3). An anatomical variation was also identified: the distal and middle third of the duct (5 cm from the duodenum) appeared duplicated. Both the duplicate channels were dilated, especially the most caudal one, which was highly tortuous with multiple narrowings and dilations of up to 7 mm. Additionally, the extra-hepatic biliary tree was also found to be dilated. The caliber of the choledochal channel was 6 mm, the common hepatic duct was 4 mm, and the left and right hepatic ducts were 3 and 2 mm, respectively. Lastly, the gallbladder was found to be distended by biliary sludge.

After considering the family history, a genetic evaluation (panel included PRSS1, PRSS2, SPINK1, CTRC, CPA1, CEL, PNLIP, PNLIPRP2, CLDN2, CFTR and CASR genes) was performed which identified the presence of a PRSS1 mutation (specifically, the familiar variant c.365G > A, p.Arg122His, exon 13).

The family was provided with multidisciplinary counseling, which included information about therapeutic possibilities based on the natural history of the pathology and the boy's risk factors. the family expressed their preference against any invasive maneuvers—even endoscopic procedures. As such, medical management was employed.

After resolving the acute episode, the boy was discharged and subsequently monitored closely (both clinically and radiologically). Laboratory tests and imaging remained stable, except for a 5-mm hypointense image in the pancreatic head, identified on the MRI at nine months follow-up. The nature of the image was unclear, and it was not possible to rule out the possibility that it was a radiological artifact.

**Figure 4:**
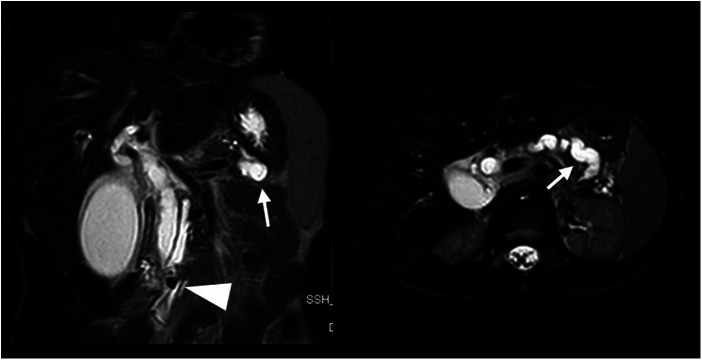
Coronal and axial T2 weighted MRI sequences performed for abdominal pain, vomiting, and loose stool demonstrates a 7 mm intraductal papillary stone (arrowhead) causing severe upstream dilatation of the biliary and pancreatic ducts, especially the lower duplicated segment of the main pancreatic duct (arrows).

A year after the previous incidence of acute pancreatitis, the patient experienced another episode. In addition to further enlargement of the gallbladder and the pancreatic and biliary ducts (up to 11 mm of maximum caliber), the imaging revealed signs of acute cholecystitis and a 7 mm calculus located in the main pancreatic duct at the level of the duodenal papilla (Figure 4). In this situation, the family agreed to the endoscopic approach. The patient underwent endoscopic-US and ERCP with Vater papilla sphincterotomy and both pancreatic (Wirsung) and biliary (common bile duct) stenting procedures. After the resolution of the acute phase, he was discharged with instructions to take ursodeoxycholic acid and scheduled for hospitalization to undergo a cholecystectomy.

One month after the episode of acute pancreatitis, just reported above, the patient returned to the Emergency Care Centre with another episode. On US, the main pancreatic duct appeared obstructed by a filling defect in the pancreatic head. The pancreatic parenchyma appeared thinned, and the biliary tree had standard caliber. We decided to perform a laparoscopic cholecystectomy, along with the removal of the pancreatic stent. After the surgery, the patient was treated medically, but his symptoms persisted and were poorly controlled by the therapy. Therefore, one week apart, he underwent ERCP (showing a filling defect between the head and body of the pancreas), dilation-assisted stone extraction (DASE), and a second pancreatic stenting procedure (7 fr, 7 cm prosthesis), which allowed the drainage of abundant dense material.

After one year, he remained asymptomatic, and the imaging and laboratory tests showed stable parameters. The functions of both the endocrine and exocrine pancreas appeared to be preserved. The duplicated pancreatic ducts progressively increased their diameter (maximum caliber 9 mm) on imaging evaluations. These ducts had an irregular course, with focal stenotic tracts and millimetric stones in the body and tail.

**Figure 5:**
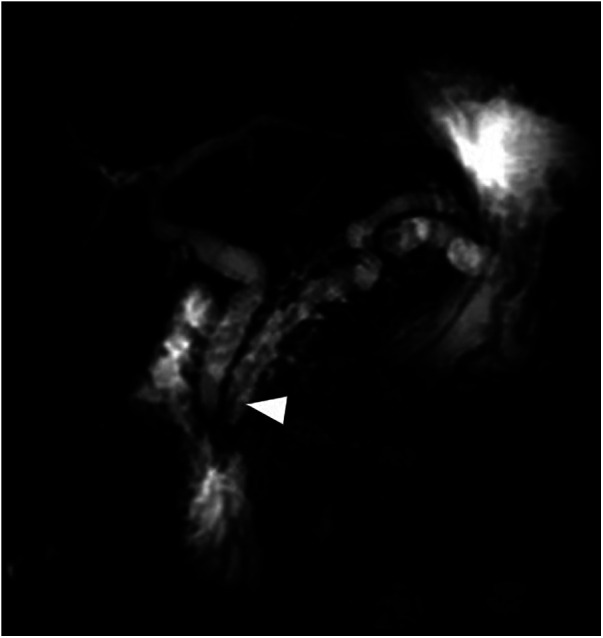
Coronal MIP reformation from MRCP performed after cholecystectomy and pancreatic duct drainage shows the stent in the main pancreatic duct (arrowhead) and persistent dilatation of the biliary and pancreatic ducts.

After 20 months, he underwent an MRI that showed ectasia of the extrahepatic biliary tract and irregularity of the pancreatic duct caliber with hypointense images (Figure 5). As a result, it was decided to perform ERCP, sphincterotomy, and removal of the pancreatic stent. During the procedure, both bifurcations of the duct could be cannulated and no stones were detected.

After being discharged in good general condition, the patient is now continuing follow-up on an outpatient basis. Although he has avoided aggressive surgery for the time being, the unique characteristics of his disease put him at a high risk of requiring pancreatic surgery in the future. A multidisciplinary team and strict follow-up have been established to allow transitional care surveillance with surgeons for adult patients.

### 2.3 Patient perspective

The patient's main goal was to be pain-free and avoid long hospitalizations, while his family initially rejected surgery and endoscopic therapeutic procedures.

## 3 Discussion

The cumulative effect of genetic and bilio-pancreatic malformations in determining recurrent and chronic pancreatitis should push us to the early identification of anatomical abnormalities in patients with more than one episode of pancreatitis or pancreatitis with a history of chronic recurrent abdominal pain.

Hereditary pancreatitis in children represents a serious illness with complex and still unclear features (3, 6, 9, 10). During childhood, It is characterized by recurring episodes of pancreatitis that can rapidly become chronic. A study conducted by the INternational Study Group of Pediatric Pancreatitis: In Search for a CuRE (INSPIRE) Cohort discovered that pathogenic PRSS1 variants, older age at the first episode of AP, or absence of toxic/metabolic risk factors increase the likelihood of developing CP, exocrine pancreatic insufficiency, and diabetes (11). Moreover, hereditary pancreatitis has a 40% cumulative risk of pancreatic ductal adenocarcinoma at 70 years of age, while the risk in the general population is only 1% (6).

Although heterogeneous, case series of patients with CP often describe the association with PD (3, 4, 6, 9, 10, 12–14). Bertin et al. tested the hypothesis of an interaction between anatomical alterations and genetics in patients with pancreatitis. They found that PD has a 47% frequency in patients with CFTR gene mutations as if these entities are cofactors associated with acute or chronic pancreatitis (3). The pathogenetic mechanism is usually represented by pancreatic duct obstruction, enzymatic activation, and elevated intra-pancreatic pressure, which are possible and common features in PD patients (13, 14). For these reasons, all the conditions that determine alterations in the drainage of pancreatic secretions are potentially causative of CP. Variations of the ductal system are quite common in the head or body of the pancreas and depend on the multiple interactions between the dorsal and ventral diverticulum during embryogenesis. The ductal anatomical variants do not usually determine pathological abnormalities. To our knowledge, this is the first case of a duplication of the distal main pancreatic duct and ductal obstruction associated with hereditary pancreatitis.

Managing patients with hereditary pancreatitis aims to control the pain, prevent recurrences, treat malfunctions (exocrine and endocrine), avoid or manage complications, and detect cancer early (14). A step-up strategy, from medical to endoscopic drainage and surgery, was advocated to reduce invasiveness (10, 14). Nevertheless, it should be remembered that endoscopic procedures, stenting, and decompressive surgical approaches permit pain relief but do not stop the inflammation or eliminate the risk of cancer (9, 15). In pediatric patients, hesitation in using surgery is often due to limited data and concerns about the invasiveness and irreversibility of some operations (10). Recently, the NASPGHAN group has provided a position paper on the role of surgery for CP children, suggesting that surgery could be considered early based on strict selection criteria (7), particularly if the patient's persistent pain is impacting their quality of life. The aspects of endoscopy and surgery are widely discussed and remain critical in the management of patients. Open questions concern the use of endoscopic preventive strategies, the timing and type of surgery required to prevent cancer.

Therapeutic ERCP is perceived as beneficial in pediatric patients with CP and should be used as a first-line treatment in uncomplicated patients (16, 17). The endoscopic approach aims to treat pancreatic ductal obstructions in case of stones or strictures and treat associated complications (16). The cross-sectional study from the INSPPIRE-2 cohort reported that the most frequent interventions are pancreatic sphincterotomy and pancreatic duct stent placement (1). However, even with complete stone removal and stricture dilatation, the recurrence of structural anomalies has been considered unavoidable (12). According to Oracz et al., pancreatic duct stenting is safe and effective (the successful completion is 98%, and the adverse event rate is reported to be 0%–17% in other series) and should be considered in patients with hereditary pancreatitis (18). In case of strictures, the stent may require removal or exchange, but the timing is not defined and is usually based on personal experience. An interval of 2–4 months has been proposed as a possible predetermined changing interval (19, 20). On the other hand, post-ERCP pancreatitis (PEP) involves 3%–16% of patients with chronic relapsing pancreatitis (21). Although specific pediatric guidelines are lacking, hydration, indomethacin, and stenting can be considered and used as preventive mechanisms (21–24). A possible limitation of pediatric endoscopic therapy, as we experienced during ERCP, is the impossibility of inserting and making the stent progress through the duct/reducing the potential for drainage. This specific challenge may be related to the patient's size or the local anatomy. The recommended patient weight for using a standard duodenoscope is ten kilograms (25). Attempts in smaller children have determined different results, and successful cannulation has been reported in 76% and 100% of cases in two recent series (26, 27). Sphincterotomy seems associated with moderate benefit and determines clinical improvement in 65% of patients (28).

In adult patients with duct stones that are equal to or greater than 5 mm, extracorporeal shockwave lithotripsy (ESWL) and pancreatoscopy are considered as alternative endotherapy approaches (22). In a series of 72 children, ESWL determined complete pain resolution in 77% of cases. Pancreatoscopy with intraductal electrohydraulic lithotripsy is preferred in cases of radiolucent stones (22).

Endoscopic ultrasound (EUS) is a diagnostic approach that images the pancreatic parenchyma, ductal anatomy, biliary risk factors, and congenital anomalies (29, 30). EUS-guided therapy is also possible for draining pancreatic collections or in cases of necrosis (22). The success rate ranges from 95 to 100%, but the pooled major adverse event rate is high (6%) (31).

The choice between endotherapy and surgery is not always straightforward. Ceppa et al. found 83% of cases of hereditary pancreatitis among 28 patients. In almost all cases (98%), an endoscopic procedure was performed, and it was successful in half of the patients, with an average duration of three years (9). The elements they considered when choosing between endotherapy and surgery included age, comorbidities, and pancreatic anatomy (duct size > 5 mm, duct stricture, stone burden). Casamassima et al. considered intractable pain the main surgical indicator and obtained a 63% success tailoring the operation to anatomy, pancreatic duct size, and genetics (10). In this context, endotherapy is a temporary “bridge strategy” for younger patients, and early referral for surgery is considered in patients with narcotic dependence.

Surgery has a role in those patients refractory to medical or endoscopic approaches and consists of conventional operations (e.g., drainage and partial resections or their combination) and total pancreatectomy with islet autotransplantation (TPIAT) (7). The decision to perform surgery should be based on a multidisciplinary evaluation and after considering a variety of factors such as symptoms and patient's debilitation, anatomy, associated genetic abnormalities and/or reversible causes of pancreatitis, response to endoscopic/medical therapy, and physiologic and psychosocial elements. Conventional operations (modified Puestow) can be considered when the main pancreatic duct is uniformly dilated to at least 7 mm and there is no inflammatory pancreatic head mass. Unfortunately, recurrences are described in up to 50% of patients at a 5-year follow-up, and the long-term outcome is poor (7, 32). Combined drainage and resection approaches could improve the drainage of the pancreatic head through the tail, while resection procedures (if possible with duodenal preservation) are used in patients with pancreatic mass. The duodenum-preserving pancreatic head resections (DPPHR, e.g., Berger, Berne, and Frey) are less invasive than the Whipple operation, which was primarily indicated for malignancies. TPIAT could have been taken into account in the management of our patient. Indeed, the presence of a genetic risk factor and the initial symptom recurrence represent strong surgical indications. Moreover, the peculiar anatomy may have hampered the success of other surgical procedures. Literature data indicates that TPIAT leads to 90% pain reduction, but it is a major operation requiring extensive demolition of the pancreas and duodenum (and often spleen), and the family was strongly against it.

Hereditary pancreatitis with anatomical abnormalities has emerged as a significant cause of the spectrum of acute, recurrent, and chronic pancreatitis in children. To determine the best endoscopic and surgical treatments, it is crucial to accurately identify the anatomical and genetic characteristics of the condition. Future directions should evaluate the impact of genetics and associated risk factors, delve into the natural history, and personalized treatments in pediatric patients. Long-term monitoring is necessary to track pain recurrence and any oncological risks, particularly during the transition from adolescence to adulthood.

## Data Availability

The original contributions presented in the study are included in the article/Supplementary Material, further inquiries can be directed to the corresponding author.
